# Cellular Systems for Colorectal Stem Cancer Cell Research

**DOI:** 10.3390/cells14030170

**Published:** 2025-01-22

**Authors:** Tatyana A. Grigoreva, Daria N. Kindt, Aleksandra V. Sagaidak, Daria S. Novikova, Vyacheslav G. Tribulovich

**Affiliations:** Laboratory of Molecular Pharmacology, St. Petersburg State Institute of Technology (Technical University), 190013 St. Petersburg, Russiatribulovich@gmail.com (V.G.T.)

**Keywords:** cancer stem cells (CSCs), spheroid, colonosphere, organoid, 3D cell culture

## Abstract

Oncological diseases consistently occupy leading positions among the most life-threatening diseases, including in highly developed countries. At the same time, the second most common cause of cancer death is colorectal cancer. The current level of research shows that the development of effective therapy, in this case, requires a new grade of understanding processes during the emergence and development of a tumor. In particular, the concept of cancer stem cells that ensure the survival of chemoresistant cells capable of giving rise to new tumors is becoming widespread. To provide adequate conditions that reproduce natural processes typical for tumor development, approaches based on increasingly complex cellular systems are being improved. This review discusses the main strategies that allow for the study of the properties of tumor cells with an emphasis on colorectal cancer stem cells. The features of working with tumor cells and the advantages and disadvantages of 2D and 3D culture systems are considered.

## 1. Introduction

Oncological diseases are one of the most acute problems of modern healthcare, causing considerable concern due to the high prevalence and mortality. Over the past few decades, significant progress has been made in reducing smoking and developing screening and therapies, primarily in the field of lung and breast cancer. However, according to 2023 data, the second most common cause of cancer death (9.3%) is colorectal cancer among all oncological diseases, inferior only to lung cancer [1].

The most common cause of death in cancer patients (more than 90%) who received both traditional chemotherapeutic agents and new targeted drugs is the development of tumor drug resistance [2]. Resistance to one chemotherapeutic drug can cause simultaneous resistance to a number of chemotherapeutic drugs with different structures and mechanisms of action. This multiple drug resistance can not only occur after a course of treatment with chemotherapeutic or targeted drugs but can also be inherent to the tumor [3,4].

Currently, multiple drug resistance is one of the main problems in the treatment of patients with various types of cancer; it is typical for colon, colorectal, breast, lung, ovary, blood cancer, and lower gastrointestinal system cancers. It also plays an important role in the development of cancer metastases and relapses [2,5]. The exact molecular mechanisms underlying the development of drug resistance in colorectal cancer cells are still unknown, but several key aspects can be identified, such as P-glycoprotein-mediated efflux, altered metabolism compared to normal cells, and the maintenance of a small group of cells with stem-like properties [5,6,7]. Chemotherapy and radiotherapy can be successful against the bulk of tumor cells but do not kill colorectal cancer stem cells, which then provide not only relapses but also tumor metastasis [4].

Cell technologies are widely used in colorectal cancer research and therapy, not only allowing new drugs to be tested but also the study of peculiarities of tumor cell functioning and the prospects for using personalized medicine. In particular, cancer stem cells (CSCs) are attractive potential targets for the treatment of colorectal cancer, but operating these cells has a number of features [8]. This review highlights the methods used to work with such cells.

## 2. The CSC Concept

It is assumed that cancer stem cells are responsible for the formation of multiple drug resistance, as well as for tumor relapse and metastasis [4]. CSCs were first isolated in human acute myeloid leukemia; these cells were able to provoke tumor development in immunodeficient mice and had a number of differences from the bulk of tumor cells [9,10]. Later, similar subpopulations were found in other types of cancer, including colorectal cancer [11,12,13].

The CSC concept suggests that tumors have a hierarchical organization similar to that of normal tissues. While the hierarchy of normal tissues is maintained by healthy stem cells, the hierarchy of tumors is maintained by a small subpopulation of cancer cells, CSCs, capable of initiating a tumor and supporting its growth [14]. In this case, CSCs form cellular heterogeneity, leading to the appearance of several different cell types in a tumor [15,16].

**Figure 1:**
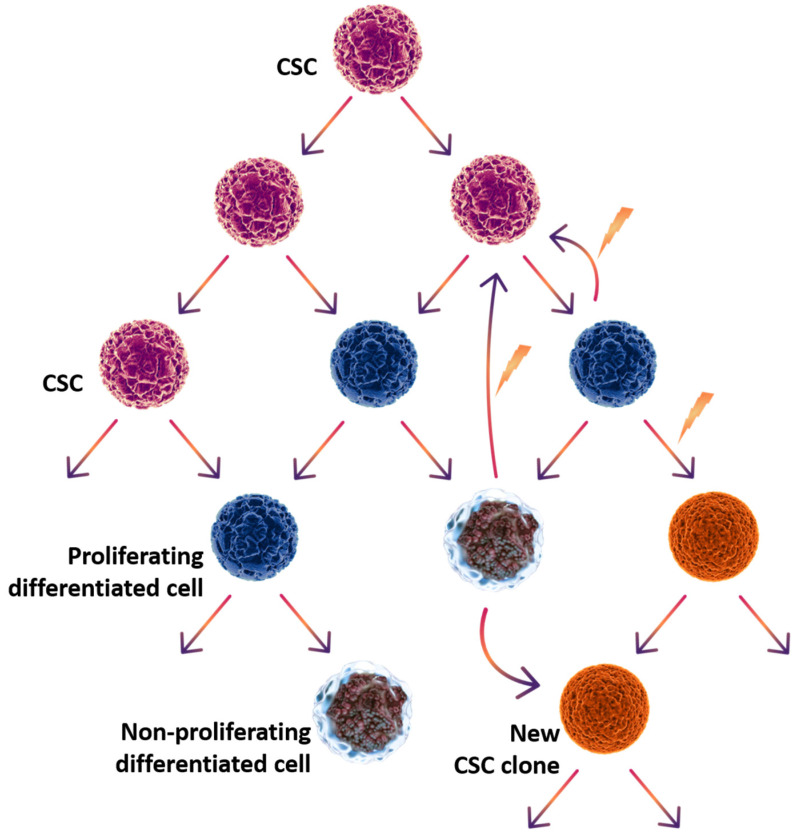
Scheme of cancer stem cell proliferation and differentiation.

CSCs can divide both symmetrically, with the formation of two new CSCs of the same clone, and asymmetrically, with the formation of CSCs and a temporarily proliferating differentiated cancer cell. The number of divisions of temporarily proliferating cancer cells is strictly limited. It is assumed that, in contrast to the differentiation of normal tissue cells, the differentiation of tumor cells can be reversible. Even terminally differentiated cells, i.e., cells incapable of further division, can dedifferentiate under certain conditions and acquire the properties of CSCs [17,18]. The stemness and plasticity of CSCs are determined by various internal and external signals, such as tumor microenvironment signals genetic or epigenetic changes. These signals, acting together or separately, affect the future of both undifferentiated and differentiated cells [14]. Thus, non-CSCs can serve as a source for the creation of CSC populations throughout oncogenesis. In addition, a mutation in the differentiated cell can provide it with the ability to self-renew and create a new clone of CSCs, adding functional diversity within the tumor [19,20]. The scheme of proliferation and differentiation of cancer stem cells is shown in Figure 1.

## 3. Specificity of Working with CSCs

As the understanding of the role of cancer stem cells in tumor functioning has developed, approaches aimed at destroying cancer stem cells, including inhibition of key CSC signaling pathways, such as the WNT or NOTCH pathways, and the use of antibody–drug conjugates targeting tumor cell stemness markers, have been proposed [21,22]. However, significant success has not been achieved yet. Difficulties in developing such therapy are associated with the characteristics of cancer stem cells, which are difficult to maintain under laboratory conditions, and with their low content in the tumor population, no more than 2% [23]. In addition, the imperfection of classical two-dimensional (2D) cell culturing systems does not allow for studying CSCs and testing new drugs aimed at targeting them [24].

The main approach to studying the activity of such cells is based on the transplantation of CSCs into laboratory animals with disabled immunity [13,25,26,27]. This poses a number of difficulties, including the selection of correct CSC markers and problems related to the dissociation of the tumor mass during transplantation [28]. In addition, the use of animals imposes a number of restrictions; from an ethical point of view, such experiments should be replaced, if possible, with more humane and effective methods of scientific research and testing. Researchers are constantly developing models for studying CSCs, using organs on a chip and 2D and 3D cultivation under specific conditions in addition to laboratory animals.

At present, a panel of markers typical for colon CSCs has been proposed; it is mainly represented by membrane glycoproteins that determine adhesion and signaling (Table 1). These markers are used both to assess the cellular composition of the tumor and to sort CSCs for subsequent inoculation into an animal or testing using FACS and MACS. However, other approaches based on the physiological characteristics of CSCs are also possible. Thus, a colony formation assay can be used to enrich the culture with CSCs since in a serum-free medium containing certain growth factors, CSCs, unlike other cells, are able to form a cell colony or sphere [29]. The low sensitivity of such cells to chemotherapy and radiotherapy can also be used for selection [30,31].

## 4. Two-Dimensional Models of Human Colorectal Cancer Cell Cultures

The in vitro cultivation of cells on a flat substrate, otherwise known as the so-called 2D culture, is a traditional method for studying cancer cells, historically used in scientific research since the early 1900s [41,42]. Primary cultures isolated directly from tumor samples are serially passaged until a homogeneous cell line is obtained [42]. To date, 2D cultures have been used in oncology research for the long-term preservation and maintenance of cell lines, as well as for various in vitro cell studies [43,44]. The advantages of this cultivation method include its availability and low cost (Table 2) [41]. The two-dimensional culture does not require expensive reagents and laboratory plastic with a specific coating. In addition, such cultures are easy to maintain and process with various chemical compounds [45].

However, 2D cultures are unable to reproduce the structure, physiology, and natural microenvironment of a tumor [54,55]. The interaction of cells with their extracellular matrix, which controls cell growth, proliferation, and function, cannot be replicated under 2D culture conditions [56]. Two-dimensional cultures do not have the characteristics of in vivo tissues; their architecture is not tissue-specific. Therefore, their behavior, in particular proliferation and response to external stimuli, differs from the behavior of real cancer cells. Two-dimensional models do not reproduce the entire complexity of cellular interactions and, accordingly, the aggressiveness and heterogeneity of the tumor [24]. Thus, 2D cultures are not able to replicate the natural functioning of CSCs.

Cells cultured in two-dimensional systems perform complex biological functions in their own way, such as cell invasion, apoptosis, transcription regulation, receptor expression, and cell proliferation [57,58]. Two-dimensional cells are unable to maintain normal morphology, have a different organization of cell surface receptors and low viability, and are more sensitive to the effects of drugs [46,47]. Cultivation in three-dimensional systems allows for overcoming the limitations of 2D cultivation and creating in vitro cultures of cancer cells that are closest in their properties to real tumors. Three-dimensional cultivation systems are currently being actively developed and used to study various types of cancer and CSCs and to test new drugs [59].

## 5. Three-Dimensional Models of Human Colorectal Cancer Cell Cultures

The first 3D cell culture model was created in 1992 by Petersen and Bissell; it imitated the natural physiological properties of breast cancer [60]. Such systems turned out to be suitable for the propagation of both normal and cancer stem cells [61]. Subsequently, similar approaches were applied to other types of tumors [11,62,63,64]. In the case of colorectal cancer, the concept of “colonosphere”, similar to “neurosphere”, “mammosphere”, etc., was used.

The sphere-formation assay has become a convenient method for the identification of CSCs, their properties, and the antitumor activity of various compounds specifically targeting CSCs since, when grown under non-adherent serum-free conditions, most tumor cells undergo anoikis (a form of programmed cell death), whereas CSCs keep dividing to form multicellular 3D spheres [44,65,66,67]. The sphere-formation assay and its recently proposed mathematical interpretation allow us to estimate the symmetric division rate of CSCs and to assess the effect of the treatment on the self-renewal and proliferative activity of these cells. This assay is a powerful tool for assessing the functional and phenotypic properties of CSCs [67].

**Figure 2:**
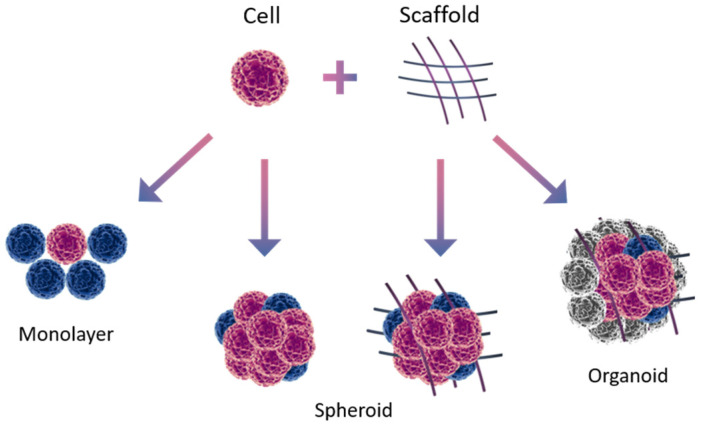
Types of cell structures used in colorectal cancer modeling.

3D systems not only solve the problems associated with traditional 2D culturing but also provide more valuable information on 3D cell–cell and cell–extracellular matrix interactions [48,49,50,68]. Three-dimensional cultures mimic the pathophysiological tumor microenvironment, provide a more clinically representative response to the tested bioactive molecules, and are therefore more adequate models for preclinical trials of various therapeutic agents in vitro [51,52,68]. In addition, 3D culturing provides a more physiologically relevant approach to the analysis of gene functions and cell phenotype ex vivo [53]. Thus, 3D systems allow for a significant reduction in the number of animal studies. In the future, 3D culturing may become an important tool for studying cellular changes, interactions, and molecular signaling during malignant transformation [51,69]. A visual comparison of 2D and 3D cultivation models is presented in Table 2 and Figure 2.

The main advantage of 3D culturing is the imitation of the extracellular matrix structure of the tissue, which is especially important for the accurate functioning of CSCs in vitro [70]. The extracellular matrix is a network of noncellular fibrous proteins, various structural macromolecules (auxiliary proteins), and adhesion molecules that provide structural and biochemical support for the cells and are necessary for many basic processes [41,71,72]. In addition, it forms cell binding sites that control their adhesion and migration [73]. From a structural point of view, the extracellular matrix consists of the interstitial connective tissue and the basement membrane. The interstitial connective tissue contains proteins, glycoproteins, and proteoglycans (polysaccharides) [70]. It consists mainly of protein molecules such as collagen I and III, self-assembling polysaccharides, and glycosaminoglycans, namely hyaluronic acid, proteoglycan, and fibronectin [41,71,72]. The basement membrane, located in normal tissues on the basal side of epithelial or endothelial cells, provides a physical barrier between the epithelial cells and the connective tissue (stroma) of the organ, allowing only gas diffusion and transport of signaling molecules [41,70].

There is currently no single technology for creating 3D cell cultures; the choice of a specific method for developing a 3D model depends on the purpose of the study. To recreate important intercellular interactions in vitro, 3D model cells are cultured in a man-made microenvironment that replicates the geometric, mechanical, and biochemical properties of the extracellular matrix of tumors in vivo [41,74]. These conditions allow the formation of 3D tissues with a structure similar to that of natural tissue [75].

3D models allow us to study changes in the morphology and cellular organization of natural tissues during oncogenic transformation [41]. Three-dimensional tumor cultures are also indispensable tools for studying in vitro mechanisms of tumor growth and metastasis [76]. The most useful for these purposes are 3D models obtained from primary human cell cultures [77].

It is worth noting that despite the numerous advantages of 3D cultures, they cannot replace 2D cultivation in the context of drug development since the productivity of such screenings decreases dramatically. In addition, the heterogeneity of the outer and inner layers of spheroids complicates the interpretation of the results.

The choice of a 3D culturing method is determined by a number of factors, such as the following:

- The nature of the grown cells and their response to the environment (cells can be selected according to a certain characteristic from the total number of cells of the corresponding strain, represent an isolated cell line of the corresponding strain or a primary culture, and can be of tissue origin);
- The available type of the artificial microenvironment in which the cells will be grown;
- The presence and available type of a scaffold based on biomaterials (natural, synthetic, or composite);
- The available type of signaling molecules (proteins and growth factors) [41,78].

Three-dimensional culturing methods (Table 3) can be divided into scaffold-based ones, which utilize natural or artificial solid scaffolds, and scaffold-free ones, which do not use them [79]. The scaffold-free approach is used to cultivate so-called spheroid cultures; the use of scaffolds allows for obtaining more complex structures: organoids [24,80]. Each 3D model used has its own advantages and disadvantages in terms of reproducing the in vivo physiology and original architecture of the tumor. Three-dimensional culturing methods are constantly being improved to better reproduce cancer biology. In particular, developments in automation, miniaturization, and adaptation of 3D models to various types of human tumors will allow active study of the antitumor immune response [24].

### 5.1. Spheroid Cultures

The formation of spheroids by cells is based on the phenomenon of self-assembly, a natural process occurring during morphogenesis and organogenesis [99]. Spheroids are solid clusters of round-shaped cells of variable size (from 50 to 150 μm) with well-balanced morphology and the ability to persist as free-floating cultures [41,99,100]. Three-dimensional spheres can be homotypic, formed by only one type of cells, or heterotypic, consisting of different types of cells [41,101].

Spheroids, due to their structure, are able to reproduce the cellular heterogeneity of solid tumors [19,102]. This ability is determined by the peculiarity of the spheroid structure formed by a necrotic core and a peripheral layer of cells [79,100]. Since there is a lack of vascularization, oxygen and nutrients poorly penetrate deep layers of the spheroids [103]. The formation of diffusion gradients of vital substances leads to the division of the spheroid into an internal part, which is a resting area with a necrotic core and an acidic pH value of the environment, and an external part consisting of actively proliferating cells [79,103]. Hypoxia of the internal part of spheroids has an indirect effect on proliferating cells, influencing the nature of their expression, similar to what is observed in the initial phase of some solid tumors in vivo [45,104]. Thus, three-dimensional spheroid models have morphological, functional, and mass transport properties similar to those of the corresponding tissue in vivo [105].

Spheroid cultures allow for studying the morphology, topography, size, organization, and invasive and metastatic potential of cancer cells, as well as the expression of proteins and genes in them [41,106,107,108]. The advantages of spheroid cultures also include simplicity, high productivity, and low cost of production [109,110]. However, these cultures also have their drawbacks. Nevertheless, when working with such cultures, it is necessary to take into account several applied aspects, including the significant requirements for the quality of operating cells, the mobility of spheroids, which complicates the assessment of their number and size, and the risks of self-disassembly of spheroids in response to changes in the content of growth factors [41,111,112,113].

#### 5.1.1. Scaffold-Free 3D Cultivation of Spheroids

Scaffold-free 3D culture methods are simpler than scaffold-based ones and are most widely used in practice to maintain an array of cells of one type [114,115,116].

Scaffold-free methods for culturing spheroid cultures include the following [41,43,80]:

- Forced floating (based on the use of nonadhesive surfaces);
- Hanging drop;
- Suspension culture (approaches based on mixing).

Most of the currently used protocols for 3D cultivation of tumor spheroids in suspension use forced floating or hanging drop methods [65].

The forced floating method is based on the use of culture plates with special coatings that prevent cell adhesion or gels whose surface prevents cells from binding to the substrate (agarose gel, pHEMA, and pluronic acid), which promotes spontaneous spheroid formation [41,105]. The forced floating method is easy to use and convenient for monitoring the formation and growth of spheroids [41]. Another advantage of this method is the possibility of automation and, therefore, suitability for high-throughput screening [105]. A disadvantage of the forced floating method is the heterogeneity of the resulting spheroids, their shape and size [41,65,81,117].

The hanging drop method allows for spheroids to be obtained in droplets of a culture medium by inverting a specially coated plate [41]. Silicones or laboratory parafilm are used as a coating, thereby increasing the curvature of the droplet surface and, as a result, accelerating cell aggregation in its center [82]. Under surface tension and gravity, the cells accumulate at the liquid–air interface, forming a single spheroid, which subsequently undergoes self-disassembly [41,85,118]. The size of the resulting spheroid can be controlled by changing the volume of the drop or the density of the cell suspension [41]. Gas exchange in spheroids obtained by this method is higher than in spheroids obtained by the forced floating method [83]. The advantages of the hanging drop method are low cost and high productivity [41]. The disadvantages of the hanging drop method include the need to transfer spheroids to other standard plates for use in cell assays [83].

Mixing-based methods for spheroid cultivation require the use of conical-bottom centrifuge tubes or a special bioreactor [41]. In the former case, cell-to-cell adhesion is maximized by centrifugation (500× *g*, 5 min) [117]. After removal of the supernatant, the cell pellet is resuspended in the culture medium to form spheroids; to enhance cell survival, the cells are preincubated for an hour [41,65,84,119].

The bioreactor for culturing spheroids by mixing consists of a centrifuge vessel and a rotating magnetic stirrer. The contents of the centrifuge vessel are continuously stirred by convection. Under these conditions, mutual cell adhesion increases, and spheroids are formed. The stirring speed must be strictly controlled since too high speed can damage the spheroid cells, and too low speed allows the cells to sink to the bottom of the vessel without forming spheroids. This method cannot be used to obtain spheroids from cells with low cohesion since they undergo apoptosis under the above conditions [41]. The disadvantages of this method include the difficulty of monitoring the formation of spheroids, and in general, this type of spheroid cultivation is less common [65,117].

Tumor spheroids can be divided into four types based on the cell origin: multicellular spheroids, tumorspheres, tissue-derived spheres, and organotypic multicellular spheroids [41].

Multicellular tumor spheroids are obtained by culturing a suspension of cancer cells under non-adhesive conditions and with a traditional culture medium depending on the cell line for 1–7 days [83,105].

Tumorspheres are obtained from single CSCs that are capable of giving rise to a sphere by dividing to form a clone of the original cell. These spheroids are well suited for culturing and studying CSCs, and the number of CSCs in tumorspheres is higher than in other types of tumor spheroids. These spheroids are obtained by culturing a suspension of cancer cells in the presence of a stem cell medium supplemented with growth factors (requires 5–7 days to 1–2 months) [41,120,121].

Endoscopic biopsy material is used to obtain tumor spheroids from tissues. Partial dissociation and induration/remodeling of the crushed cancer tissue in a normal medium with the addition of FBS leads to the formation of spheroids (requires from 2 to 5 days to 12–18 days) [41,120,122].

Organotypic multicellular spheroids can also be obtained as a result of partial dissociation (carried out mechanically or enzymatically) of tumor tissue under non-adhesive conditions. These conditions lead to a more rapid formation of spheroids (1–3 days) [41,122].

#### 5.1.2. Scaffold-Based 3D Cultivation of Spheroids

Scaffold-based methods of 3D cultivation are based on the use of special supports of both organic and inorganic nature [123]. Currently, more than 100 types of such structures are used [41]. It is difficult to isolate the optimal scaffold material since there is currently no unified system for assessing their impact on the specific behavior of CSCs; however, each has its own advantages and disadvantages, which are discussed below.

Various natural (collagen, gelatin, elastin, silk fibroin, chitosan, chitin, fibrin, fibrinogen, etc.) and synthetic polymers, as well as composites of natural and synthetic substances, are used as scaffolds for 3D cultivation. The composites used imitate the natural extracellular matrix in terms of porosity, fibrousness, permeability, and mechanical stability. The microarchitecture of the scaffolds improves the biophysical and biochemical interaction of adherent cells, which allows for their better expression in vitro. Thus, scaffolds for 3D cultivation create a biologically active environment for cell proliferation and differentiation, as well as for the secretion of a cell-specific extracellular matrix [41,123].

Hydrogels are popular scaffolds for 3D cultivation [41]. Hydrogels consist of hydrophilic polymers linked to each other by ionic or covalent bonds, and water fills the space between the polymer chains [124,125]. Hydrogels can be natural, synthetic, and hybrid, combining natural and synthetic components [86,126]. The main advantage of hydrogels over other scaffolds for 3D cultivation is the ability to regulate their physicochemical properties. This feature allows for the imitation of both the biochemical and mechanical properties of the native extracellular matrix of cultured cells [41]. Another advantage of hydrogels is that they provire cells with good access to nutrition and oxygen, transmitted by diffusion even into fairly deep layers [126].

Agarose hydrogels are thought to be the easiest-to-use materials for obtaining 3D cultures of various cell types [41]. Changing the composition, concentration, and volume of the gel allows for the creation of optimal conditions for each specific cell type [123].

The type I collagen scaffold is often used for 3D cultivation [41]. The advantages of this scaffold are its ease of use, low cost, and facile adaptation to solve various research problems. These structural properties of the gel, such as pore size, ligand density, and rigidity can be varied quite easily by changing the concentration of collagen or using chemical cross-linking compounds [41,127,128,129].

One of the most popular ready-made natural scaffolds for 3D culturing is the reconstituted basement membrane isolated from mouse Engelbert–Holmes–Roy sarcoma. It is a liquid mixture of gelatinous proteins that turns into a gel at 37 °C [87,130]. This material produces a large amount of extracellular matrix rich in type I collagen, laminin-111, heparan sulfate proteoglycan (perlecan), and nidogen and also contains soluble growth factors such as fibroblast growth factor (FGF), epidermal growth factor (EGF), transforming growth factor-β (TGF-β), and matrix metalloproteinases (MMPs), including MMP-2 and MMP-9 [41,131]. Due to this biological activity, the gel ensures the formation of complex three-dimensional structures consisting of several cell types under normal cultivation conditions and is widely used to obtain various 3D cultures in vitro. It also allows for the evaluation of the migration and behavior of cancer cells, serving as a widely available model for studying many fundamental issues of cell biology [41,88,89,132]. The disadvantages of the material due to its animal origin include the heterogeneity of the composition, which varies from batch to batch, while uncontrolled changes in the content of growth factors, up to the appearance of endogenous growth factors in the composition that are not typical for human tumor environment, can have a multidirectional, unpredictable effect on the results of the studies [44,68,133]. In addition, it is necessary to take into account that at 4 °C, the gel becomes liquid, which complicates the work when varying the temperature [79]. Given these disadvantages, many researchers prefer to use hydrogel systems since they themselves do not contain growth factors and, accordingly, the concentration of added growth factors can be easily controlled [41,68].

Along with natural hydrogels, synthetic hydrogels based on polyethylene glycol (PEG), polyvinyl alcohol, or poly-2-hydroxyethyl methacrylate are also used to obtain 3D cultures [41]. These scaffolds consist of synthetic organic polymers and represent a model of an extracellular matrix with clearly defined characteristics and are actively used for 3D cultivation of nerve, bone, cartilage, muscle and kidney cells [41,90,91,92,134,135,136]. The popularity of scaffolds based on synthetic hydrogels is due to the simplicity and ease of use, as well as good reproducibility of properties between batches [41,90,91,92,134,135,136,137,138].

The most significant drawback of synthetic hydrogels is the lack of basal biological activity. These scaffolds are not capable of transmitting biochemical signals that determine the fate of cells [41,138]. The problem of the lack of basal biological activity can be solved by adding signaling molecules: peptides, growth factors, and glycans [139]. However, biomolecules in synthetic hydrogels can be distributed unevenly. In addition, these scaffolds have problems with oxygen access for cells, the heterogeneity of the synthetic cellular microenvironment, and the release of toxic hydrogel degradation products into cultured cells [41,137,140]. The use of scaffolds based on polylactic acid, polyglycolic acid, and their copolymer polylactide-co-glycolide (PLGA) is difficult due to their biodegradation, accompanied by the release of degradation products such as lactic acid [141]. It is also known that the rigidity of PEG-based gels and the cross-linking density of their constituent polymers greatly influence the growth, morphology, proliferation, and migration of cells [41,93,136].

Synthetic scaffolds for 3D culturing of cells in vitro are created based not only on hydrogels but also on PLGA or PLGA-PEG polymer nanofibers [41]. Nanofibers are produced by various methods, such as electrogrinding, phase separation, and self-assembly, and can have different chemical and mechanical properties, including diameter, length, and porosity [94,142]. Being randomly arranged, they create a thin sheet. Nanofiber-based scaffolds have several significant advantages over hydrogel-based scaffolds. Nanofibers not only form the basis for the extracellular matrix but also provide cancer cells with the necessary topographic conditions for the formation of 3D cultures. In addition, the resulting tumor cell cultures are easy to visualize. These advantages can make nanofiber-based scaffolds convenient and effective tools for testing various drugs [41].

### 5.2. Organoid Structures

Organoids are a recent advance in 3D modeling of tumors in vitro and are quite different from spheroids [24]. They are represented by complex clusters of cells specific to organs such as the stomach, liver, bladder, and intestine [80]. Organoids are composed of stem or progenitor cells and self-organize when provided with an extracellular scaffolding environment. Given the right conditions, organoids grow into microscopic versions of their parent organs, mimicking their architecture, functionality, and genetic features [143,144]. Organoid cultures are derived from adult stem cells, endothelial stem cells, and induced pluripotent stem cells.

The first organoid cultures were obtained from adult stem cells [80]. A method for obtaining colorectal cancer organoid cultures was first developed by Sato et al. [145]. The researchers selected long-term culturing conditions for individual intestinal crypts or single stem cells containing leucine-rich repeats in G protein-coupled receptors (LGR5+) obtained from the mouse small intestine. The use of growth factors and a scaffold that mimics the in vivo environment of stem cells allowed them to obtain unlimitedly growing organotypic, highly polarized epithelial structures with proliferative crypts and differentiated villous compartments from a single stem cell [145,146]. The developed protocol was subsequently used to grow healthy colon, liver, pancreas, and prostate tissues, as well as malignant tissues, which allowed them to study oncogenic mutations in patients in vitro [95,96,97,139,145].

Tumor organoids can be generated from induced pluripotent stem cells [80]. The development of this method for generating tumor organoids relies on the CRISPR/Cas9 genome editing method, which can induce desired oncogenic mutations in normal healthy adult stem cells obtained from a patient [147]. In theory, epithelial cancer stem cells can be reprogrammed to become pluripotent and used to generate tumor organoids [80]. However, in practice, this task appears to be difficult since genetic and epigenetic abnormalities in cancer can interfere with cell differentiation trajectories [148]. Some researchers believe that it is more practical to generate tumor organoids directly from patient-derived cancer cells without the intermediate step of inducing pluripotency [143].

A particularly important advantage of organoid cultures is their long-term preservation of phenotypic and genetic similarity to the tumor epithelium of patients [149]. In addition, organoids allow one to take into account the interactions of cancer cells with stromal and immune cells and, therefore, are excellent models for testing new drugs in vitro [143,150]. Organoids obtained from the tissues of cancer patients imitate the organization of the tumor in vivo at the histopathological, molecular, and functional levels and can predict the drug response of patients to the treatment [151]. Organoid cultures can also be used to analyze the mechanisms of drug resistance in tumors [24].

Despite significant advantages, organoid cultures have a number of disadvantages. One of them is the difference in phenotypes of cultured organoids, which arises due to their self-organizing structure and interferes with drug screening [24]. Various bioengineering techniques are used to standardize the morphology of organoids, including cultivation using various scaffolds [98]. Another important disadvantage of organoid cultures is the growth rate, which can take several weeks [151,152]. In addition, organoids cannot fully replicate the complexity of a tumor in vivo since they lack a number of epithelial components, tumor stroma, and microbiome [153].

The possibility of organoid cryopreservation has led to the development of living biobanks of organoids from healthy and tumor tissues [80]. A biobank of organoids from 20 patients with colorectal cancer was created by van de Wetering et al. and contains both tumor organoids and organoids from adjacent healthy tissues [154]. This biobank allows for the study of oncogenic genetic diversity in colorectal cancer and for high-throughput screening of drugs used to identify the relationship between tumor genotype and drug efficacy, which is especially important for the development of targeted drugs aimed at a specific patient, i.e., for personalized medicine [80].

## 6. Microfluidic Systems for Colorectal Cancer Research

The next step towards bringing cell cultures and in vivo systems closer together is the creation of the so-called “organ-on-a-chip” models. These systems are significantly more complex than the approaches described above, but they allow for the reproduction of biochemical and biophysical conditions, including vascularization [155,156,157,158,159].

In the case of colorectal cancer, cells obtained from the corresponding tumor lines or from patients are cultured in a microfluidic system [158,159,160]. Due to the inclusion of physical forces and vascular components in the system, such models demonstrate greater transcriptional similarity to the tumor tissue compared with 3D models [160,161] and allow for a reduction in the number of animal studies [159]. Such features could improve the predictive ability of models in the development of antitumor drugs, including within the framework of personalized medicine approaches, and in studies of processes accompanying tumor development, including metastasis [158,162]. However, the labor intensity of the methods limits their application compared with the use of various colonospheres.

## 7. Conclusions and Future Perspectives

In recent years, cell technologies have been developed significantly, allowing us to study various aspects of cellular existence, starting from monoclonal cultures, which provide the opportunity to deeply investigate specific mechanisms, and ending with the creation of organs-on-a-chip, which reproduce the influence of the microenvironment. At the same time, it is not possible to unequivocally discard any of the approaches as outdated and irrelevant. The increasing complexity of cellular systems brings their reactions closer to a real organism. Moreover, the dramatic discrepancy between the reactions of specific cell cultures in vitro and the real processes of multicellular organisms is becoming increasingly obvious. It was repeatedly shown that it is the combination of factors and the environment that determines the reactions of cells, including tumor cells, to external factors. In the case of colorectal cancer, the nature of the reactions is also determined by the gut microbiota in addition to the cellular heterogeneity of the “host”, tumor microenvironment, and the specificity of CSCs [153,160,163]. However, bringing closer to a real organism also elaborates research, which discards researchers back to the use of less “correct” but fundamentally simpler systems for screening biologically active molecules, including the use of artificially induced chemoresistant systems [164,165].

Thus, 3D systems provide more opportunities for studying CSCs, including colorectal ones and developing new drugs targeting CSCs compared with 2D culture systems. Three-dimensional colorectal cancer cell culturing is the most biologically representative tool for studying CSCs and evaluating the efficacy of new drugs in vitro. Three-dimensional systems open new prospects for studying oncogenesis and developing more effective drugs and also reduce the number of animal studies.

## Figures and Tables

**Table 1:** Stemness markers used for the identification and selection of colorectal CSCs.

Marker	Localization	Reference
CD24	Transmembrane glycoprotein	[32]
CD44	Transmembrane glycoprotein	[33]
CD133	Transmembrane glycoprotein	[34]
CD166	Transmembrane glycoprotein	[35]
CD326	Transmembrane glycoprotein	[36]
ALDH1	Intracellular enzyme	[37]
SOX2	Intracellular transcription factor	[38]
OCT4	Intracellular transcription factor	[39]
STAT3	Intracellular transcription factor	[40]

**Table 2:** Comparison of 2D and 3D cultivation models [24,41,44,46,47,48,49,50,51,52,53].

Characteristics	2D	3D	Native State
Available interactions	Cell–cell	Cell–cell and cell–matrix	Cell–cell and cell–matrix
Impact of environment	Homogeneous	Heterogeneous	Heterogeneous
Cell shape	Flat and oblate	Natural structure	Natural structure
Intercellular communication	+/−	+	+
Cell differentiation	+/−	+	+
Cell proliferation rate	++	+	+
Cell sensitivity to treatment	+++	++	+
Suitability for CSCs	+/−	+	+
Ease of operation	+	+/−	−
Cost	+/−	++	−

“+/−”—the characteristic is weakly expressed or limitedly applicable to the considered model; “+”, “++”, “+++”—the characteristic is normally/intensively/strongly expressed in the considered model; “–”—the characteristic is not applicable to the considered model.

**Table 3:** Overview of methods for 3D culturing.

3D Model	Scaffold	Method	References
Spheroid	Scaffold-free	Forced floating	[63,64,65,66,67,81]
		Hanging drop	[82,83]
		Mixing	[84]
	Scaffold-based	Culturing on natural scaffolds	[65,85,86,87,88,89]
		Culturing on artificial scaffolds	[52,71,90,91,92,93,94]
Organoid	Scaffold-based	Culturing on natural scaffolds	[95,96,97]
		Culturing on artificial scaffolds	[98]

## Data Availability

No new data were created or analyzed in this study. Data sharing is not applicable to this article.

## References

[1] Bray F., Laversanne M., Sung H., Ferlay J., Siegel R.L., Soerjomataram I., Jemal A. (2024). Global cancer statistics 2022: GLOBOCAN estimates of incidence and mortality worldwide for 36 cancers in 185 countries. CA Cancer J. Clin..

[2] Bukowski K., Kciuk M., Kontek R. (2020). Mechanisms of multidrug resistance in cancer chemotherapy. Int. J. Mol. Sci..

[3] Abdallah H.M., Al-Abd A.M., El-Dine R.S., El-Halawany A.M. (2015). P-glycoprotein inhibitors of natural origin as potential tumor chemo-sensitizers: A review. J. Adv. Res..

[4] Chen L., Yang F., Chen S., Tai J. (2022). Mechanisms on chemotherapy resistance of colorectal cancer stem cells and research progress of reverse transformation: A mini-review. Front. Med..

[5] Ashique S., Bhowmick M., Pal R., Khatoon H., Kumar P., Sharma H., Garg A., Kumar S., Das U. (2024). Multi drug resistance in Colorectal Cancer- approaches to overcome, advancements and future success. Adv. Cancer Biol. Metastasis.

[6] Grigoreva T., Vorona S., Novikova D., Tribulovich V. (2022). Analysis of P-glycoprotein transport cycle reveals a new way to identify efflux inhibitors. ACS Omega..

[7] Grigoreva T., Sagaidak A., Novikova D., Tribulovich V. (2022). Implication of ABC transporters in non-proliferative diseases. Eur. J. Pharmacol..

[8] Hervieu C., Christou N., Battu S., Mathonnet M. (2021). The role of cancer stem cells in colorectal cancer: From the basics to novel clinical trials. Cancers.

[9] Bonnet D., Dick J.E. (1997). Human acute myeloid leukemia is organized as a hierarchy that originates from a primitive hematopoietic cell. Nat. Med..

[10] Lapidot T., Sirard C., Vormoor J., Murdoch B., Hoang T., Caceres-Cortes J., Minden M., Paterson B., Caligiuri M.A., Dick J.E. (1994). A cell initiating human acute myeloid leukaemia after transplantation into SCID mice. Nature.

[11] Ricci-Vitiani L., Lombardi D.G., Pilozzi E., Biffoni M., Todaro M., Peschle C., De Maria R. (2007). Identification and expansion of human colon-cancer-initiating cells. Nature.

[12] O’Brien C.A., Pollett A., Gallinger S., Dick J.E. (2007). A human colon cancer cell capable of initiating tumour growth in immunodeficient mice. Nature.

[13] Dalerba P., Dylla S.J., Park I.K., Liu R., Wang X., Cho R.W., Hoey T., Gurney A., Huang E.H., Simeone D.M. (2007). Phenotypic characterization of human colorectal cancer stem cells. Proc. Natl. Acad. Sci. USA.

[14] Prasetyanti P.R., Medema J.P. (2017). Intra-tumor heterogeneity from a cancer stem cell perspective. Mol. Cancer.

[15] Clevers H. (2011). The cancer stem cell: Premises, promises and challenges. Nat. Med..

[16] Huang T., Song X., Xu D., Tiek D., Goenka A., Wu B., Sastry N., Hu B., Cheng S.Y. (2020). Stem cell programs in cancer initiation, progression, and therapy resistance. Theranostics.

[17] Medema J.P. (2013). Cancer stem cells: The challenges ahead. Nat. Cell Biol..

[18] Meacham C.E., Morrison S.J. (2013). Tumour heterogeneity and cancer cell plasticity. Nature.

[19] De Sousa E Melo F., Vermeulen L., Fessler E., Medema J.P. (2013). Cancer heterogeneity—A multifaceted view. EMBO Rep..

[20] Campbell L.L., Polyak K. (2007). Breast tumor heterogeneity: Cancer stem cells or clonal evolution?. Cell Cycle.

[21] Takebe N., Miele L., Harris P.J., Jeong W., Bando H., Kahn M., Yang S.X., Ivy S.P. (2015). Targeting Notch, Hedgehog, and Wnt pathways in cancer stem cells: Clinical update. Nat. Rev. Clin. Oncol..

[22] Laszlo G.S., Estey E.H., Walter R.B. (2014). The past and future of CD33 as therapeutic target in acute myeloid leukemia. Blood Rev..

[23] Yang L., Shi P., Zhao G., Xu J., Peng W., Zhang J., Zhang G., Wang X., Dong Z., Chen F. (2020). Targeting cancer stem cell pathways for cancer therapy. Signal Transduct. Target. Ther..

[24] Ramzy G.M., Koessler T., Ducrey E., McKee T., Ris F., Buchs N., Rubbia-Brandt L., Dietrich P.Y., Nowak-Sliwinska P. (2020). Patient-derived in vitro models for drug discovery in colorectal carcinoma. Cancers.

[25] De Angelis M.L., Zeuner A., Policicchio E., Russo G., Bruselles A., Signore M., Vitale S., de Luca G., Pilozzi E., Boe A. (2016). Cancer stem cell-based models of colorectal cancer reveal molecular determinants of therapy resistance. Stem Cells Transl. Med..

[26] Lv J., Liu Y., Mo S., Zhou Y., Chen F., Cheng F., Li C., Saimi D., Liu M., Zhang H. (2022). Gasdermin E mediates resistance of pancreatic adenocarcinoma to enzymatic digestion through a YBX1-mucin pathway. Nat. Cell Biol..

[27] Lee J.S., Eo P., Kim M.C., Kim J.B., Jin H.K., Bae J.S., Jeong J.H., Park H.Y., Yang J.D. (2019). Effects of Stromal Vascular Fraction on Breast Cancer Growth and Fat Engraftment in NOD/SCID Mice. Aesthetic Plast. Surg..

[28] Batlle E., Clevers H. (2017). Cancer stem cells revisited. Nat. Med..

[29] Taylor M.D., Poppleton H., Fuller C., Su X., Liu Y., Jensen P., Magdaleno S., Dalton J., Calabrese C., Board J. (2005). Radial glia cells are candidate stem cells of ependymoma. Cancer Cell.

[30] Chiodi I., Belgiovine C., Donà F., Scovassi A.I., Mondello C. (2011). Drug treatment of cancer cell lines: A way to select for cancer stem cells?. Cancers.

[31] Bhaskara V.K., Mohanam I., Rao J.S., Mohanam S. (2012). Intermittent hypoxia regulates stem-like characteristics and differentiation of neuroblastoma cells. PLoS ONE.

[32] Nosrati A., Naghshvar F., Maleki I., Salehi F. (2016). Cancer stem cells CD133 and CD24 in colorectal cancers in Northern Iran. Gastroenterol. Hepatol. Bed Bench.

[33] Jing F., Kim H.J., Kim C.H., Kim Y.J., Lee J.H., Kim H.R. (2015). Colon cancer stem cell markers CD44 and CD133 in patients with colorectal cancer and synchronous hepatic metastases. Int. J. Oncol..

[34] Glumac P.M., LeBeau A.M. (2018). The role of CD133 in cancer: A concise review. Clin. Transl. Med..

[35] Shafaei S., Sharbatdaran M., Kamrani G., Khafri S. (2013). The association between CD166 detection rate and clinicopathologic parameters of patients with colorectal cancer. Casp. J. Intern. Med..

[36] Tseng J.Y., Yang C.Y., Yang S.H., Lin J.K., Lin C.H., Jiang J.K. (2015). Circulating CD133^+^/ESA^+^ cells in colorectal cancer patients. J. Surg. Res..

[37] Holah N.S., Aiad H.A., Asaad N.Y., Elkhouly E.A., Lasheen A.G. (2017). Evaluation of the role of ALDH1 as cancer stem cell marker in colorectal carcinoma: An immunohistochemical study. J. Clin. Diagn. Res..

[38] Lundberg I.V., Edin S., Eklöf V., Öberg Å., Palmqvist R., Wikberg M.L. (2016). SOX2 expression is associated with a cancer stem cell state and down-regulation of CDX2 in colorectal cancer. BMC Cancer.

[39] Bu X., Liu Y., Wang L., Yan Z., Xin G., Su W. (2023). Oct4 promoted proliferation, migration, invasion, and epithelial-mesenchymal transition (EMT) in colon cancer cells by activating the SCF/c-Kit signaling pathway. Cell Cycle.

[40] Lai Z., Wang H., Tang X., Zhang L., Wang T., Cheng J. (2022). Study on the mechanism of diosgenin targeting STAT3 to inhibit colon cancer proliferation and migration. Dis. Markers.

[41] Habanjar O., Diab-Assaf M., Caldefie-Chezet F., Delort L. (2021). 3D Cell Culture Systems: Tumor Application, Advantages, and Disadvantages. Int. J. Mol. Sci..

[42] Becker J.L., Blanchard D.K. (2007). Characterization of primary breast carcinomas grown in three-dimensional cultures. J. Surg. Res..

[43] Breslin S., O’Driscoll L. (2013). Three-dimensional cell culture: The missing link in drug discovery. Drug Discov. Today.

[44] Amaral R.L.F., Miranda M., Marcato P.D., Swiech K. (2017). Comparative analysis of 3D bladder tumor spheroids obtained by forced floating and hanging drop methods for drug screening. Front. Physiol..

[45] Grigoreva T., Sagaidak A., Vorona S., Novikova D., Tribulovich V. (2022). The ATP mimetic attack on the nucleotide-binding domain to overcome ABC transporter mediated chemoresistance. ACS Med. Chem. Lett..

[46] Costa E.C., Moreira A.F., de Melo-Diogo D., Gaspar V.M., Carvalho M.P., Correia I.J. (2016). 3D tumor spheroids: An overview on the tools and techniques used for their analysis. Biotechnol. Adv..

[47] Hutchinson L., Kirk R. (2011). High drug attrition rates--where are we going wrong?. Nat. Rev. Clin. Oncol..

[48] Bray L.J., Binner M., Holzheu A., Friedrichs J., Freudenberg U., Hutmacher D.W., Werner C. (2015). Multi-parametric hydrogels support 3D in vitro bioengineered microenvironment models of tumour angiogenesis. Biomaterials.

[49] Debnath J., Brugge J.S. (2005). Modelling glandular epithelial cancers in three-dimensional cultures. Nat. Rev. Cancer.

[50] Kievit F.M., Florczyk S.J., Leung M.C., Wang K., Wu J.D., Silber J.R., Ellenbogen R.G., Lee J.S.H., Zhang M. (2014). Proliferation and Enrichment of CD133^+^ Glioblastoma Cancer Stem Cells on 3D Chitosan-Alginate Scaffolds. Biomaterials.

[51] Langhans S.A. (2018). Three-Dimensional in Vitro Cell Culture Models in Drug Discovery and Drug Repositioning. Front. Pharmacol..

[52] Fiore D., Di Giacomo F., Kyriakides P., Inghirami G. (2017). Patient-Derived-Tumor-Xenograft: Modeling cancer for basic and translational cancer research. Clin. Diagn. Pathol..

[53] Moysidou C.-M., Barberio C., Owens R.M. (2021). Advances in Engineering Human Tissue Models. Front. Bioeng. Biotechnol..

[54] Petersen O.W., Rønnov-Jessen L., Howlett A.R., Bissell M.J. (1992). Interaction with Basement Membrane Serves to Rapidly Distinguish Growth and Differentiation Pattern of Normal and Malignant Human Breast Epithelial Cells. Proc. Natl. Acad. Sci. USA.

[55] Dolznig H., Walzl A., Kramer N., Rosner M., Garin-Chesa P., Hengstschläger M. (2011). Organotypic spheroid cultures to study tumor–stroma interaction during cancer development. Drug Discov. Today Dis. Models.

[56] Svendsen C.N., ter Borg M.G., Armstrong R.J.E., Rosser A.E., Chandran S., Ostenfeld T., Caldwell M.A. (1998). A new method for the rapid and long term growth of human neural precursor cells. J. Neurosci. Methods.

[57] Dontu G., Abdallah W.M., Foley J.M., Jackson K.W., Clarke M.F., Kawamura M.J., Wicha M.S. (2003). In vitro propagation and transcriptional profiling of human mammary stem/progenitor cells. Genes Dev..

[58] Ponti D., Costa A., Zaffaroni N., Pratesi G., Petrangolini G., Coradini D., Pilotti S., Pierotti M.A., Diadone M.G. (2005). Isolation and in vitro propagation of tumorigenic breast cancer cells with stem/progenitor cell properties. Cancer Res..

[59] Bahmad H.F., Cheaito K., Chalhoub R.M., Hadadeh O., Monzer A., Ballout F., El-Hajj A., Mukherji D., Liu Y.N., Daoud G. (2018). Sphere-Formation Assay: Three-Dimensional in vitro Culturing of Prostate Cancer Stem/Progenitor Sphere-Forming Cells. Front. Oncol..

[60] Shaheen S., Ahmed M., Lorenzi F., Nateri A.S. (2016). Spheroid-Formation (Colonosphere) Assay for in Vitro Assessment and Expansion of Stem Cells in Colon Cancer. Stem Cell Rev. Rep..

[61] Morrison B.J., Steel J.C., Morris J.C. (2012). Sphere culture of murine lung cancer cell lines are enriched with cancer initiating cells. PLoS ONE.

[62] Redmond J., Mccarthy H., Buchanan P., Levingstone T.J., Dunne N.J. (2021). Advances in biofabrication techniques for collagen-based 3D in vitro culture models for breast cancer research. Mater. Sci. Eng. C.

[63] Do Amaral R.J.F.C., Zayed N.M.A., Pascu E.I., Murphy C.M., Sridharan R., González-Vázquez A., Sullivan B.O. (2019). Functionalising collagen-based scaffolds with platelet-rich plasma for enhanced skin wound healing potential. Front. Bioeng. Biotechnol..

[64] Li Y., Huang G., Li M., Wang L., Elson E.L., Lu T.J., Genin G.M., Xu F. (2016). An approach to quantifying 3D responses of cells to extreme strain. Sci. Rep..

[65] Lee G.Y., Kenny P.A., Lee E.H., Bissell M.J. (2007). Three-dimensional culture models of normal and malignant breast epithelial cells. Nat. Methods.

[66] Yamada K.M., Cukierman E. (2007). Modeling tissue morphogenesis and cancer in 3D. Cell.

[67] Lei Y., Schaffer D.V. (2013). A fully defined and scalable 3D culture system for human pluripotent stem cell expansion and differentiation. Proc. Natl. Acad. Sci. USA.

[68] Wang F., Weaver V.M., Petersen O.W., Larabell C.A., Dedhar S., Briand P., Lupu R., Bissell M.J. (1998). Reciprocal interactions between beta1-integrin and epidermal growth factor receptor in three-dimensional basement membrane breast cultures: A different perspective in epithelial biology. Proc. Natl. Acad. Sci. USA.

[69] Muthuswamy S.K., Li D., Lelievre S., Bissell M.J., Brugge J.S. (2001). ErbB2, but Not ErbB1, Reinitiates proliferation and induces luminal repopulation in epithelial acini. Nat. Cell Biol..

[70] Poltavets V., Kochetkova M., Pitson S.M., Samuel M.S. (2018). The Role of the extracellular matrix and its molecular and cellular regulators in cancer cell plasticity. Front. Oncol..

[71] Sawicki L.A., Choe L.H., Wiley K.L., Lee K.H., Kloxin A.M. (2018). Isolation and identification of proteins secreted by cells cultured within synthetic hydrogel-based matrices. ACS Biomater. Sci. Eng..

[72] Malik R., Lelkes P.I., Cukierman E. (2015). Biomechanical and biochemical remodeling of stromal extracellular matrix in cancer. Trends Biotechnol..

[73] Brown N.H. (2011). Extracellular matrix in development: Insights from mechanisms conserved between invertebrates and vertebrates. Cold Spring Harb. Perspect. Biol..

[74] Holen I., Nutter F., Wilkinson J.M., Evans C.A., Avgoustou P., Ottewell P.D. (2015). Human breast cancer bone metastasis in vitro and in vivo: A novel 3D model system for studies of tumour cell-bone cell interactions. Clin. Exp. Metastasis.

[75] Egeblad M., Nakasone E.S., Werb Z. (2010). Tumors as Organs: Complex Tissues that interface with the entire organism. Dev. Cell.

[76] Sethi T., Rintoul R.C., Moore S.M., MacKinnon A.C., Salter D., Choo C., Chilvers E.R., Dransfield I., Donnelly S.C., Strieter R. (1999). Extracellular matrix proteins protect small cell lung cancer cells against apoptosis: A mechanism for small cell lung cancer growth and drug resistance in Vivo. Nat. Med..

[77] Espinoza-Sánchez N.A., Chimal-Ramírez G.K., Fuentes-Pananá E.M. (2018). Analyzing the communication between monocytes and primary breast cancer cells in an extracellular matrix extract (ECME)-based three-dimensional system. JoVE.

[78] Moroni L., Burdick J.A., Highley C., Lee S.J., Morimoto Y., Takeuchi S., Yoo J.J. (2021). Biofabrication strategies for 3D in vitro models and regenerative medicine. Nat. Rev. Mater..

[79] Knight E., Przyborski S. (2015). Advances in 3D cell culture technologies enabling tissue-like structures to be created in vitro. J. Anat..

[80] Ramos P., Carvalho M.R., Chen W., Yan L.P., Zhang C.H., He Y.L., Reis R.L., Oliveira J.M. (2023). Microphysiological systems to study colorectal cancer: State-of-the-art. Biofabrication.

[81] Amann A., Zwierzina M., Gamerith G., Bitsche M., Huber J.M., Vogel G.F., Blumer M., Koeck S., Pechriggl E.J., Kelm J.M. (2014). Development of an Innovative 3D Cell Culture System to Study Tumour-Stroma Interactions in Non-Small Cell Lung Cancer Cells. PLoS ONE.

[82] Godugu C., Patel A.R., Desai U., Andey T., Sams A., Singh M. (2013). AlgiMatrixTM Based 3D cell culture system as an in-vitro tumor model for anticancer studies. PLoS ONE.

[83] Costa E.C., Gaspar V.M., Coutinho P., Correia I.J. (2014). Optimization of liquid overlay technique to formulate heterogenic 3D co-cultures models. Biotechnol. Bioeng..

[84] Mueller-Klieser W. (2000). Tumor biology and experimental therapeutics. Crit. Rev. Oncol. Hematol..

[85] Thurber G.M., Schmidt M.M., Wittrup K.D. (2008). Antibody tumor penetration: Transport opposed by systemic and antigen-mediated clearance. Adv. Drug Deliv. Rev..

[86] Shield K., Ackland M.L., Ahmed N., Rice G.E. (2009). Multicellular spheroids in ovarian cancer metastases: Biology and pathology. Gynecol. Oncol..

[87] Friedrich J., Seidel C., Ebner R., Kunz-Schughart L.A. (2009). Spheroid-based drug screen: Considerations and practical approach. Nat. Protoc..

[88] Ekert J.E., Johnson K., Strake B., Pardinas J., Jarantow S., Perkinson R., Colter D.C. (2014). Three-dimensional lung tumor microenvironment modulates therapeutic compound responsiveness in vitro—Implication for drug development. PLoS ONE.

[89] Hongisto V., Jernström S., Fey V., Mpindi J.-P., Kleivi Sahlberg K., Kallioniemi O., Perälä M. (2013). High-throughput 3D screening reveals differences in drug sensitivities between culture models of JIMT1 breast cancer cells. PLoS ONE.

[90] Zhang Y.S., Duchamp M., Oklu R., Ellisen L.W., Langer R., Khademhosseini A. (2016). bioprinting the cancer microenvironment. ACS Biomater. Sci. Eng..

[91] Caliari S.R., Burdick J.A. (2016). A Practical guide to hydrogels for cell culture. Nat. Methods.

[92] Laschke M.W., Menger M.D. (2017). Life Is 3D: Boosting spheroid function for tissue engineering. Trends Biotechnol..

[93] Singec I., Knoth R., Meyer R.P., Maciaczyk J., Volk B., Nikkhah G., Frotscher M., Snyder E.Y. (2006). Defining the actual sensitivity and specificity of the neurosphere assay in stem cell biology. Nat. Methods..

[94] Sarisozen C., Dhokai S., Tsikudo E.G., Luther E., Rachman I.M., Torchilin V.P. (2016). Nanomedicine based curcumin and doxorubicin combination treatment of glioblastoma with ScFv-targeted micelles: In vitro evaluation on 2D and 3D tumor models. Eur. J. Pharm. Biopharm..

[95] Wang X., Zhen X., Wang J., Zhang J., Wu W., Jiang X. (2013). Doxorubicin delivery to 3D multicellular spheroids and tumors based on boronic acid-rich chitosan nanoparticles. Biomaterials.

[96] Lin R.Z., Chang H.Y. (2008). Recent advances in three-dimensional multicellular spheroid culture for biomedical research. Biotechnol. J..

[97] Jaganathan H., Gage J., Leonard F., Srinivasan S., Souza G.R., Dave B., Godin B. (2014). Three-dimensional in vitro co-culture model of breast tumor using magnetic levitation. Sci. Rep..

[98] Ryu N.-E., Lee S.-H., Park H. (2019). Spheroid culture system methods and applications for mesenchymal stem cells. Cells.

[99] Costa E.C., de Melo-Diogo D., Moreira A.F., Carvalho M.P., Correia I.J. (2018). Spheroids formation on non-adhesive surfaces by liquid overlay technique: Considerations and practical approaches. Biotechnol. J..

[100] Achilli T.-M., Meyer J., Morgan J.R. (2012). Advances in the formation, use and understanding of multi-cellular spheroids. Expert. Opin. Biol. Ther..

[101] Rasouli M., Safari F., Kanani M.H., Ahvati H. (2024). Principles of hanging drop method (spheroid formation) in cell culture. Methods in Molecular Biology.

[102] Tran C., Kalra V. (2013). Fabrication of porous carbon nanofibers with adjustable pore sizes as electrodes for supercapacitors. J. Power Sources.

[103] Phipps M.C., Clem W.C., Grunda J.M., Clines G.A., Bellis S.L. (2012). Increasing the pore sizes of bone-mimetic electrospun scaffolds comprised of polycaprolactone, collagen I and hydroxyapatite to enhance cell infiltration. Biomaterials.

[104] Kelm J.M., Timmins N.E., Brown C.J., Fussenegger M., Nielsen L.K. (2003). Method for generation of homogeneous multicellular tumor spheroids applicable to a wide variety of cell types. Biotechnol. Bioeng..

[105] Li J., He F., Pei M. (2011). Creation of an in Vitro microenvironment to enhance human fetal synovium-derived stem cell chondrogenesis. Cell Tissue Res..

[106] Carpenedo R.L., Sargent C.Y., McDevitt T.C. (2007). Rotary suspension culture enhances the efficiency, yield, and homogeneity of embryoid body differentiation. Stem Cells.

[107] Smart C.E., Morrison B.J., Saunus J.M., Vargas A.C., Keith P., Reid L., Wockner L., Amiri M.A., Sarkar D., Simpson P.T. (2013). In Vitro analysis of breast cancer cell line tumourspheres and primary human breast epithelia mammospheres demonstrates inter- and intrasphere heterogeneity. PLoS ONE.

[108] Yu M., Bardia A., Aceto N., Bersani F., Madden M.W., Donaldson M.C., Deasi R., Zhu H., Comaills V., Zheng Z. (2014). Cancer Therapy. Ex Vivo culture of circulating breast tumor cells for individualized testing of drug susceptibility. Science.

[109] Kondo J., Endo H., Okuyama H., Ishikawa O., Iishi H., Tsujii M., Ohue M., Inoue M. (2011). Retaining cell-cell contact enables preparation and culture of spheroids composed of pure primary cancer cells from colorectal cancer. Proc. Natl. Acad. Sci. USA.

[110] Ravi M., Paramesh V., Kaviya S.R., Anuradha E., Solomon F.D. (2015). 3D cell culture systems: Advantages and applications. J. Cell. Physiol..

[111] Elisseeff J. (2008). Hydrogels: Structure starts to gel. Nat. Mater..

[112] Slaughter B.V., Khurshid S.S., Fisher O.Z., Khademhosseini A., Peppas N.A. (2009). Hydrogels in regenerative medicine. Adv. Mater..

[113] Martínez-Ramos C., Lebourg M. (2015). Three-dimensional constructs using hyaluronan cell carrier as a tool for the study of cancer stem cells. J. Biomed. Mater. Res. Part B Appl. Biomater..

[114] Hamdi D.H., Barbieri S., Chevalier F., Groetz J.-E., Legendre F., Demoor M., Galera P., Lefaix J.-L., Saintigny Y. (2015). In Vitro engineering of human 3D chondrosarcoma: A preclinical model relevant for investigations of radiation quality impact. BMC Cancer.

[115] Baker E.L., Bonnecaze R.T., Zaman M.H. (2009). Extracellular matrix stiffness and architecture govern intracellular rheology in cancer. Biophys. J..

[116] Baker E.L., Srivastava J., Yu D., Bonnecaze R.T., Zaman M.H. (2011). Cancer cell migration: Integrated roles of matrix mechanics and transforming potential. PLoS ONE.

[117] Harjanto D., Zaman M.H. (2011). Matrix mechanics and receptor–ligand interactions in cell adhesion. Org. Biomol. Chem..

[118] Mouhieddine T.H., Nokkari A., Itani M.M., Chamaa F., Bahmad H., Monzer A., El-Merahbi R., Daoud G., Eid A., Kobeissy F.H. (2015). Metformin and ara-a effectively suppress brain cancer by targeting cancer stem/progenitor cells. Front. Neurosci..

[119] Mulfaul K., Giacalone J.C., Voigt A.P., Riker M.J., Ochoa D., Han I.C., Stone E.M., Mullins R.F., Tucker B.A. (2020). Stepwise differentiation and functional characterization of human induced pluripotent stem cell-derived choroidal endothelial cells. Stem Cell Res. Ther..

[120] Benton G., Kleinman H.K., George J., Arnaoutova I. (2011). Multiple uses of basement membrane-like matrix (BME/Matrigel) In Vitro and In Vivo with cancer cells. Int. J. Cancer.

[121] Arnaoutova I., George J., Kleinman H.K., Benton G. (2009). The Endothelial Cell Tube Formation Assay on Basement Membrane Turns 20: State of the Science and the Art. Angiogenesis.

[122] Dolega M.E., Abeille F., Picollet-D’hahan N., Gidrol X. (2015). Controlled 3D culture in matrigel microbeads to analyze clonal acinar development. Biomaterials.

[123] Fridman R., Giaccone G., Kanemoto T., Martin G.R., Gazdar A.F., Mulshine J.L. (1990). Reconstituted basement membrane (matrigel) and laminin can enhance the tumorigenicity and the drug resistance of small cell lung cancer cell lines. Proc. Natl. Acad. Sci. USA.

[124] Manuscript A. (2009). Modular extracellular matrices: Solutions for the puzzle. Methods.

[125] Lutolf M.P., Hubbell J.A. (2005). Synthetic biomaterials as instructive extracellular microenvironments for morphogenesis in tissue engineering. Nat. Biotechnol..

[126] Murphy A.R., Laslett A., O’Brien C.M., Cameron N.R. (2017). Scaffolds for 3D In Vitro culture of neural lineage cells. Acta Biomater..

[127] Burdick J.A., Anseth K.S. (2002). Photoencapsulation of osteoblasts in injectable RGD-modified PEG hydrogels for bone tissue engineering. Biomaterials.

[128] Bryant S.J., Durand K.L., Anseth K.S. (2003). Manipulations in hydrogel chemistry control photoencapsulated chondrocyte behavior and their extracellular matrix production. J. Biomed. Mater. Res. Part A.

[129] Adelöw C., Segura T., Hubbell J.A., Frey P. (2008). The Effect of Enzymatically Degradable Poly(Ethylene Glycol) Hydrogels on Smooth Muscle Cell Phenotype. Biomaterials.

[130] Strutz F., Zeisberg M., Renziehausen A., Raschke B., Becker V., van Kooten C., Müller G. (2001). TGF-Beta 1 induces proliferation in human renal fibroblasts via induction of basic Fibroblast Growth Factor (FGF-2). Kidney Int..

[131] Fang Y., Eglen R.M. (2017). Three-dimensional cell cultures in drug discovery and development. SLAS Discov..

[132] Zhang N., Milleret V., Thompson-Steckel G., Huang N.-P., Vörös J., Simona B.R., Ehrbar M. (2017). Soft hydrogels featuring in-depth surface density gradients for the simple establishment of 3D tissue models for screening applications. SLAS Discov. Adv. Life Sci. RD.

[133] Brown T.E., Anseth K.S. (2017). Spatiotemporal hydrogel biomaterials for regenerative medicine. Chem. Soc. Rev..

[134] Sell S.A., Wolfe P.S., Garg K., McCool J.M., Rodriguez I.A., Bowlin G.L. (2010). The Use of Natural Polymers in Tissue Engineering: A Focus on Electrospun Extracellular Matrix Analogues. Polymers.

[135] Ouyang A., Ng R., Yang S.-T. (2007). Long-term culturing of undifferentiated embryonic stem cells in conditioned media and three-dimensional fibrous matrices without extracellular matrix coating. Stem Cells.

[136] Bryant S.J., Anseth K.S., Lee D.A., Bader D.L. (2004). Crosslinking density influences the morphology of chondrocytes photoencapsulated in PEG hydrogels during the application of compressive strain. J. Orthop. Res..

[137] Girard Y.K., Wang C., Ravi S., Howell M.C., Mallela J., Alibrahim M., Green R., Hellermann G., Mohapatra S.S., Mohapatra S. (2013). A 3D fibrous scaffold inducing tumoroids: A platform for anticancer drug development. PLoS ONE.

[138] Karuri N.W., Liliensiek S., Teixeira A.I., Abrams G., Campbell S., Nealey P.F., Murphy C.J. (2004). Biological length scale topography enhances cell-substratum adhesion of human corneal epithelial cells. J. Cell Sci..

[139] Drost J., Clevers H. (2018). Organoids in cancer research. Nat. Rev. Cancer.

[140] Lou Y.-R., Leung A. (2018). Next generation organoids for biomedical research and applications. Biotechnol. Adv..

[141] Sato T., Stange D.E., Ferrante M., Vries R.G., Van Es J.H., Van den Brink S., Van Houdt W.J., Pronk A., Van Gorp J., Siersema P.D. (2011). Long-term expansion of epithelial organoids from human colon, adenoma, adenocarcinoma, and Barrett’s epithelium. Gastroenterology.

[142] Pimenta J., Ribeiro R., Almeida R., Costa P.F., da Silva M.A., Pereira B. (2022). Organ-on-chip approaches for intestinal 3D In Vitro modeling. Cell. Mol. Gastroenterol. Hepatol..

[143] Mun S.J., Ryu J.S., Lee M.O., Son Y.S., Oh S.J., Cho H.S., Son M.Y., Kim D.S., Kim S.J., Yoo H.J. (2019). Generation of expandable human pluripotent stem cell-derived hepatocyte-like liver organoids. J. Hepatol..

[144] Drost J., Karthaus W.R., Gao D., Driehuis E., Sawyers C.L., Chen Y., Clevers H. (2016). Organoid culture systems for prostate epithelial and cancer tissue. Nat. Protoc..

[145] Broutier L., Andersson-Rolf A., Hindley C.J., Boj S.F., Clevers H., Koo B.K., Huch M. (2016). Culture and establishment of self-renewing human and mouse adult liver and pancreas 3D organoids and their genetic manipulation. Nat. Protoc..

[146] Driehuis E., Clevers H. (2017). CRISPR/Cas 9 genome editing and its applications in organoids. Am. J. Physiol. Gastrointest. Liver Physiol..

[147] Idris M., Alves M.M., Hofstra R.M.W., Mahe M.M., Melotte V. (2021). Intestinal multicellular organoids to study colorectal cancer. Biochim. Biophys. Acta Rev. Cancer.

[148] Quevedo R., Smirnov P., Tkachuk D., Ho C., El-Hachem N., Safikhani Z., Pugh T.J., Haibe-Kains B. (2020). Assessment of genetic drift in large pharmacogenomic studies. Cell Syst..

[149] Stein W.D., Litman T., Fojo T., Bates S.E. (2004). A Serial Analysis of Gene Expression (SAGE) database analysis of chemosensitivity: Comparing solid tumors with cell lines and comparing solid tumors from different tissue origins. Cancer Res..

[150] Vlachogiannis G., Hedayat S., Vatsiou A., Jamin Y., Fernández-Mateos J., Khan K., Lampis A., Eason K., Huntingford I., Burke R. (2018). Patient-derived organoids model treatment response of metastatic gastrointestinal cancers. Science.

[151] Lancaster M.A., Corsini N.S., Wolfinger S., Gustafson E.H., Phillips A., Burkard T.R., Otani T., Livesey F.J., Knoblich J.A. (2017). Guided self-organization and cortical plate formation in human brain organoids. Nat. Biotechnol..

[152] Lancaster M.A., Huch M. (2019). Disease modelling in human organoids. Dis. Model. Mech..

[153] Sasaki N., Clevers H. (2018). Studying cellular heterogeneity and drug sensitivity in colorectal cancer using organoid technology. Curr. Opin. Genet. Dev..

[154] Van de Wetering M., Francies H.E., Francis J.M., Bounova G., Iorio F., Pronk A., van Houdt W., van Gorp J., Taylor-Weiner A., Kester L. (2015). Prospective derivation of a living organoid biobank of colorectal cancer patients. Cell.

[155] Hachey S.J., Movsesyan S., Nguyen Q.H., Burtin-Sojo G., Tankazyan A., Wu J., Hoang T., Zhao D., Wang S., Hatch M.M. (2021). An in vitro vascularized micro-tumor model of human colorectal cancer recapitulates in vivo responses to standard-of-care therapy. Lab Chip.

[156] Sobrino A., Phan D.T., Datta R., Wang X., Hachey S.J., Romero-López M., Gratton E., Lee A.P., George S.C., Hughes C.C. (2016). 3D microtumors in vitro supported by perfused vascular networks. Sci. Rep..

[157] Shirure V.S., Hughes C.C.W., George S.C. (2021). Engineering Vascularized Organoid-on-a-Chip Models. Annu. Rev. Biomed. Eng..

[158] Carvalho M.R., Barata D., Teixeira L.M., Giselbrecht S., Reis R.L., Oliveira J.M., Truckenmüller R., Habibovic P. (2019). Colorectal tumor-on-a-chip system: A 3D tool for precision onco-nanomedicine. Sci. Adv..

[159] Petreus T., Cadogan E., Hughes G., Smith A., Pilla Reddy V., Lau A., O’Connor M.J., Critchlow S., Ashford M., Oplustil O’Connor L. (2021). Tumour-on-chip microfluidic platform for assessment of drug pharmacokinetics and treatment response. Commun. Biol..

[160] Strelez C., Perez R., Chlystek J.S., Cherry C., Yoon A.Y., Haliday B., Shah C., Ghaffarian K., Sun R.X., Jiang H. (2023). Integration of patient-derived organoids and organ-on-chip systems: Investigating colorectal cancer invasion within the mechanical and GABAergic tumor microenvironment. bioRxiv.

[161] Park S.E., Georgescu A., Huh D. (2019). Organoids-on-a-Chip. Science.

[162] Strelez C., Chilakala S., Ghaffarian K., Lau R., Spiller E., Ung N., Hixon D., Yoon A.Y., Sun R.X., Lenz H.J. (2021). Human colorectal cancer-on-chip model to study the microenvironmental influence on early metastatic spread. Iscience.

[163] Brusnic O., Onisor D., Boicean A., Hasegan A., Ichim C., Guzun A., Chicea R., Todor S.B., Vintila B.I., Anderco P. (2024). Fecal microbiota transplantation: Insights into colon carcinogenesis and immune regulation. J. Clin. Med..

[164] Grigoreva T., Sagaidak A., Novikova D., Tribulovich V. (2024). New insights into chemoresistance mediated by Mdm2 inhibitors: The benefits of targeted therapy over common cytostatics. Biomedicines.

[165] Grigoreva T., Sagaidak A., Romanova A., Novikova D., Garabadzhiu A., Tribulovich V. (2021). Establishment of drug-resistant cell lines under the treatment with chemicals acting through different mechanisms. Chem. Biol. Interact..

